# Predictors for temporary stomas non-closure among non-metastatic rectal cancer patients undergoing curative resection: a retrospective analysis

**DOI:** 10.1186/s12957-024-03403-8

**Published:** 2024-05-07

**Authors:** Chia-Chien Hsu, Wen-Sy Tsai, Tzong-yun Tsai, Jeng-Fu You, Chien-Yuh Yeh, Pao-Shiu Hsieh, Reiping Tang, Shu-Huan Huang

**Affiliations:** 1 Division of Colon and Rectal Surgery, Colorectal Section, Department of Surgery Chang, Gung Memorial Hospital, Linko, No.5, Fuxing St., Guishan Dist, Taoyuan City, 33305 Taiwan; 2 College of Medicine, Chang Gung University, Taoyuan, Taiwan

## Abstract

**Background:**

The primary treatment for non-metastatic rectal cancer is curative resection. However, sphincter-preserving surgery may lead to complications. This study aims to develop a predictive model for stoma non-closure in rectal cancer patients who underwent curative-intent low anterior resection.

**Methods:**

Consecutive patients diagnosed with non-metastatic rectal cancer between January 2005 and December 2017, who underwent low anterior resection, were retrospectively included in the Chang Gung Memorial Foundation Institutional Review Board. A comprehensive evaluation and analysis of potential risk factors linked to stoma non-closure were performed.

**Results:**

Out of 956 patients with temporary stomas, 10.3% (*n* = 103) experienced non-closure primarily due to cancer recurrence and anastomosis-related issues. Through multivariate analysis, several preoperative risk factors significantly associated with stoma non-closure were identified, including advanced age, anastomotic leakage, positive nodal status, high preoperative CEA levels, lower rectal cancer presence, margin involvement, and an eGFR below 30 mL/min/1.73m2. A risk assessment model achieved an AUC of 0.724, with a cutoff of 2.5, 84.5% sensitivity, and 51.4% specificity. Importantly, the non-closure rate could rise to 16.6% when more than two risk factors were present, starkly contrasting the 3.7% non-closure rate observed in cases with a risk score of 2 or below (*p* < 0.001).

**Conclusion:**

Prognostic risk factors associated with the non-closure of a temporary stoma include advanced age, symptomatic anastomotic leakage, nodal status, high CEA levels, margin involvement, and an eGFR below 30 mL/min/1.73m2. Hence, it is crucial for surgeons to evaluate these factors and provide patients with a comprehensive prognosis before undergoing surgical intervention.

## Background

Curative resection was considered the primary treatment approach for non-metastatic rectal cancer. Advances in treatment options, such as the introduction of neoadjuvant therapy [1], and the desire of patients to preserve anal function have shifted the preference towards sphincter-sparing surgery over abdominoperineal resection for rectal cancer patients [2, 3]. However, sphincter-preserving surgery can be accompanied by complications such as bowel dysfunction or anastomosis leakage [4]. Therefore, temporary fecal diversion is often necessary following sphincter-sparing surgery [5, 6]. The Clinical Practice Guidelines for Ostomy Surgery recommended fecal diversion as an effective method that can reduce the severity of anastomotic dehiscence [7].

Previous research found that 3% to 23.2% of patients who underwent sphincter-preserving surgery still required permanent stomas. [8–12] Several predisposing factors may lead to irreversible stomas, including local recurrence and distant metastasis [9, 11, 13, 14]. Other correlated factors included anastomosis leakage [12, 15], advanced age [16], male gender [9, 17], renal dysfunction [18], and elevated preoperative serum carcinoembryonic antigen (CEA) level [19]. Additionally, whether neoadjuvant chemoradiation therapy is a risk factor for permanent stoma in rectal cancer patients remains controversial [20].

This study aims to identify the presented risk factors associated with initially intended temporary stomas that ultimately did not undergo closure in non-metastatic rectal cancer patients who underwent curative-intent low anterior resection. This information can be utilized to inform the patients about the potentially unfavorable outcomes before the operation.

## Methods

**Fig. 1:**
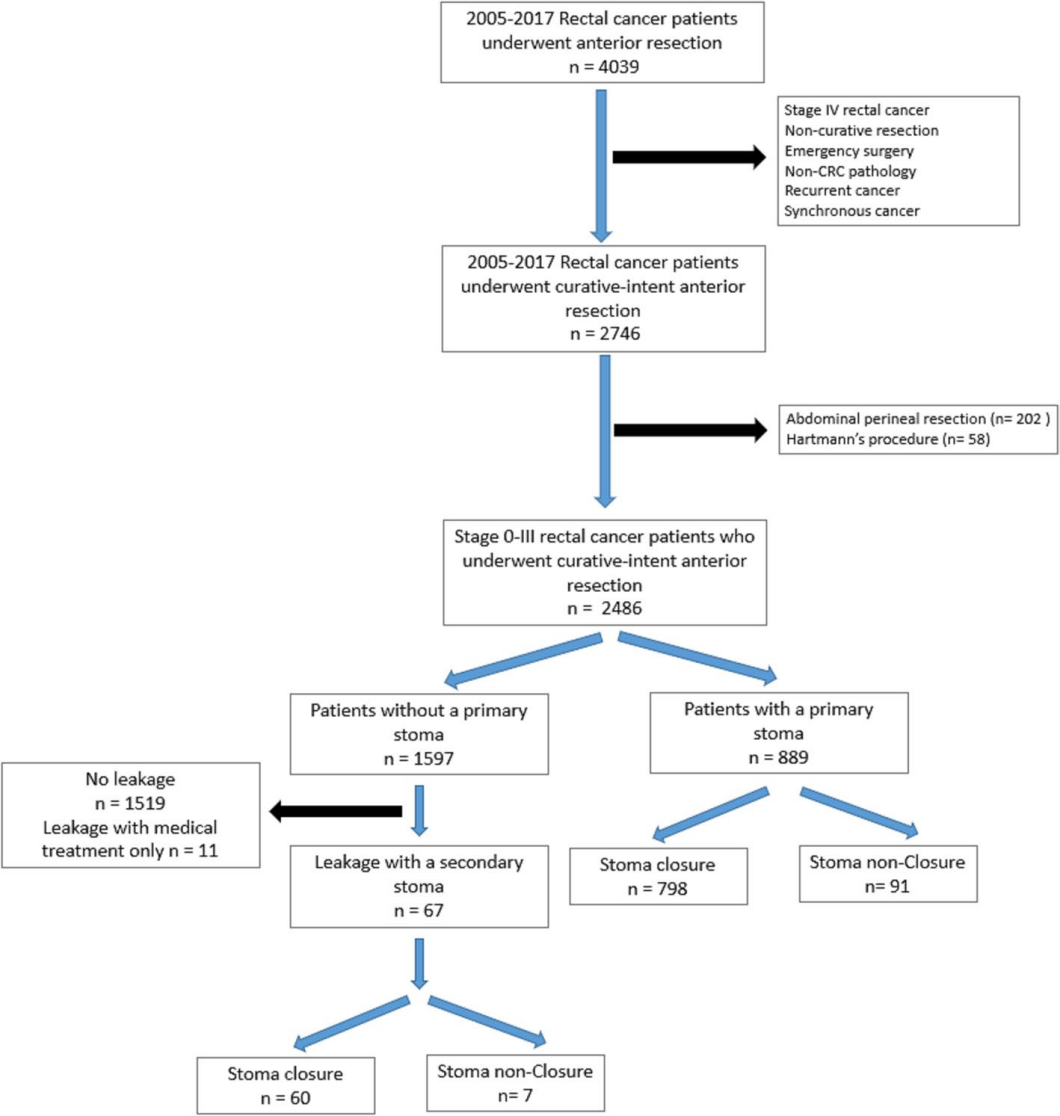
Study Population. This figure analyzes a total of 956 rectal cancer patients who underwent curative-intent low anterior resection and temporary stoma. This includes 889 patients with a primary stoma and 67 with a stoma secondary to symptomatic leakage, analyzed after exclusions

Approval number 202001577B0 was obtained from the Chang Gung Medical Foundation Institutional Review Board to conduct this study. From January 1, 2005, to December 31, 2017, consecutive patients with rectal cancer were recruited from the Division of Colorectal Surgery at Chang Gung Memorial Hospital in Linkou (Fig. 1).

This study enrolled 4039 rectal cancer patients who received surgical resection from 2005–2017. The mean follow-up time was 62.3 months, with a maximum of 134 months. The study included non-metastatic rectal cancer patients and those who achieved a pathological complete response (ypT0) following neoadjuvant treatments and underwent curative-intent resection with a stoma. Patients with stage IV rectal cancer, non-curative resection, emergency surgery, non-CRC pathology (squamous carcinoma, melanoma, or *gastrointestinal stromal tumor*), and recurrent or synchronous cancer were all excluded. For patients who received abdominal perineal resection and Hartmann's procedure were also excluded due to the challenges and limitations of stoma reversal.

Before the surgery, patients underwent preoperative evaluations, including computed tomography of the chest, abdomen, and pelvis, magnetic resonance imaging of the pelvis, preoperative serum carcinoembryonic antigen (CEA) testing, and complete colonoscopy.

Patients with locally advanced mid-to-low rectal cancer with cT3, cT4, or positive cN stage were treated with neoadjuvant concurrent chemoradiation (CCRT) or short-course radiotherapy at the discretion of the surgeon. Long-course neoadjuvant CCRT patients were administered a 5-fluoropyrimidine-based regimen (intravenous 5-fluorouracil or oral capecitabine, tegafur) and 50.4 Gy in total 28 fractions of radiotherapy, followed by surgery 6–8 weeks after the completion of treatment. Short-course radiotherapy patients received a dose of 25 Gy to the pelvis and tumor in total 5 fractions, and surgery was settled 8–10 days after the end of radiotherapy.

For locally advanced rectal cancer patients, the adjuvant chemotherapy was primarily based on 5-fluoropyrimidine (5-fluorouracil, capecitabine, or tegafur) and mainly administered within 2 months after the operation, with a total treatment course lasting 6 months. The addition of oxaliplatin was at the discretion of the surgeon. Digital rectal examinations were routinely performed to evaluate the condition of the anastomosis. An additional colonoscopy or lower gastrointestinal series would be conducted if the integrity of the anastomosis was uncertain.

After the operation, postoperative follow-up evaluations were conducted based on a standardized protocol, which included physical examinations and serum CEA level assessments every 3 months in the first 2 years, followed by assessments every 6 months from the third to the fifth year post-operation. Additionally, colonoscopies and computed tomography scans were arranged annually for the first 5 years after the operation. Additional positron emission tomography scans were arranged if preoperative or postoperative imaging reports were equivocal.

A primary stoma was defined as a stoma made during primary surgery. Loop colostomy and loop ileostomy are two types of stomas of choice, and the decision to create one was at the surgeon's discretion. Factors influencing the decision to create a preventive stoma included a narrow pelvic cavity, malnutrition status, lower rectal cancer, adverse events during surgery, or the patient's inability to tolerate anastomotic leakage. Patients who received neoadjuvant radiotherapy alone or combined chemoradiation were more likely to receive a preventive stoma at the surgeon's discretion. On the other hand, a secondary stoma was defined as a stoma created after the primary surgery due to complications such as diffuse peritonitis, medically uncontrolled pelvic sepsis, purulent discharge from the anus, fecal discharge from drainage tubes, or rectovaginal fistula. A stoma non-closure was defined as failing to perform stoma reversal surgery by the end of the follow-up period.

In this study, local recurrence was defined as recurrence within the pelvis. In contrast, distal metastasis was defined as recurrence outside the pelvis, in other organs such as the liver, lung, or bone, or non-regional lymph nodes like the para-aortic lymph nodes.

The statistical analysis was conducted using SPSS software (Version 24.0. Armonk, NY: IBM Corp). Continuous variables were compared using independent sample t-tests, while categorical variables were compared using chi-square or Fisher's exact tests, as appropriate. Multivariate logistic regression analysis, using the Allen-Cady modified backward selection procedure, was used to identify independent predictors of non-closure of the stoma. Variables with a *p*-value < 0.1 in the initial analysis were included in the multivariate model. A *p*-value < 0.05 was considered statistically significant. Receiver operating characteristic (ROC) curves were constructed to identify risk factors for the stoma's non-closure and determine the optimal cut-off points. The Kaplan–Meier method analyzed stoma-free survival in temporary stoma patients with different risk factors. The log-rank test was used to determine if there were significant differences in survival between groups (*p* < 0.05).

## Results

A total of 4039 rectal cancer patients were included in this study. After exclusion, 2486 rectal cancer patients who underwent curative-intent anterior resection were analyzed (Fig. 1). Out of these, 889 received a primary loop ileostomy or loop colostomy. Among the 1597 patients who did not initially receive a stoma, 67 later had a secondary stoma due to symptomatic leakage. Table 1 represents the characteristics between patients with a primary stoma and those without, the former was significantly more likely male, had a higher incidence of T3 stage cancers, lower rectal cancer with less than 5 cm from the anal verge, hypoalbuminemia with serum albumin levels below 3.5 g/dL, and tend to receive Neoadjuvant radiotherapy (RT) (31.5 vs. 3.4%, *p* < 0.001).

**Table 1:** Clinicopathological factors of patients with and without a primary stoma

	**Primary stoma**	**No primary stoma**	***P* value**
**Case number**	889	1597	
**Age (Std. dev)**	61.6 (12.7)	62.8 (12.3)	0.023
**Sex (%)**			< 0.001
**M**	595 (66.9)	892 (55.9)	
**F**	294 (33.1)	705 (44.1)	
**BMI (Std. dev)**	24.2 (3.6)	24.5 (10.0)	0.451
**eGFR ≤ 30** mL/min/1.73m^2^(%)	20 (2.2)	26 (1.6)	0.270
**Anastomosis leakage**	31 (3.5)	78 (4.9)	0.103
**CVA (%)**	27 (3)	43 (2.7)	0.670
**Liver cirrhosis (%)**	12 (1.3)	18 (1.1)	0.673
T stage (%)			< 0.001
0–2	557 (34.9)	251 (28.2)	
3	901 (56.4)	577 (64.9)	
4	139 (8.7)	61 (6.9)	
**N stage (%)**			0.920
**0**	484 (54.4)	883 (55.3)	
**1**	245 (27.6)	431 (27.0)	
**2**	160 (18.0)	283 (17.7)	
**Low rectal tumor (%)**	388 (43.6)	84 (5.3)	< 0.001
**Involved circumferential margin (%)**	289 (32.5)	553 (34.6)	0.097
**Laparoscopy (%)**	271 (30.5)	519 (32.5)	0.301
**Albumin < 3.5 g/dL (%)**	58 (6.5)	49 (3.1)	< 0.001
**CEA > 5** ng/mL	205 (23.1)	401 (25.1)	0.254
**Neoadjuvant RT**^a^** (%)**	280 (31.5)	55 (3.4)	< 0.001
**Adjuvant chemotherapy**	261 (29.4)	531 (33.2)	0.046

*BMI* body mass index, *eGFR* estimated Glomerular filtration rate, *CVA* Cerebrovascular accident, *CEA* carcinoembryonic antigen, *RT* radiotherapy

^a^including both long-course and short-course radiation therapy

Table 2 describes the pattern of the 103 patients who did not receive stoma reversal. Cancer recurrence (35%) and anastomosis-related complications (22.3%) made up the majority. Table 3 revealed that patients with their stoma unreversed processed a higher proportion of renal dysfunction which was reflected by eGFR ≤ 30 mL/min/1.73m^2^ (1.8% vs. 8.7%, *p* < 0.001). This group also showed a greater incidence of anastomotic leakage (21.4% vs. 8.9%, *p* < 0.001). Additionally, T3 and T4 stages patients were less likely to have their stomas reversed (*p* = 0.014), and a significantly higher proportion of patients with N1 and N2 stages were in the stoma non-closure group (29.1% vs. 27.3% and 30.1% vs. 16.2%, *p* = 0.001). Tumors involving circumferential margins and preoperative serum CEA levels above 5 ng/mL were also risks for stoma reversing (49.5% vs. 30.3%, 38.8% vs. 21.5%, *p* < 0.001).

**Table 2:** Associated disorders in individuals with a non-closure stoma

	**Case**	**percentage**
Cancer recurrence	36	35%
Other disease related	20	19.4%
Stroke	3	
Pneumonia	4	
Type A aneurysm	1	
PPU	1	
Small bowel perforation	1	
Ischemic bowel	3	
Dementia	2	
Adhesion ileus	4	
Gall bladder IAI	1	
Stoma Varices	1	0.9%
Anastomosis related	23	22.3%
Stricture	1	
Stenosis	10	
Fistula	4	
Abscess	2	
Leakage	6	
Patient related	15	14.6%
Old age	3	
Bed ridden	3	
Patient refuse	3	
Lost to follow-up	4	
Loose anal tone	2	
Unknown	8	7.8%

**Table 3:** Clinicopathological factors of patients with and without a temporary stoma closure

	**Stoma Closure**	**Stoma Non-closure**	***P* value**
**Case number**	853	103	
**Age (Std. dev)**	61.2 (12.5)	64.5 (13.4)	0.014
**Sex (%)**			0.277
**M**	576 (67.5)	75 (72.8)	
**F**	277 (32.5)	28 (27.2)	
**BMI (Std. dev)**	24.3 (3.4)	24.3 (4.0)	0.932
**eGFR ≤ 30 mL/min/1.73m**^**2**^**(%)**	15 (1.8)	9 (8.7)	< 0.001
**Anastomosis leakage**	76 (8.9)	22 (21.4)	< 0.001
**Stomy timing**			0.750
**Primary**	794 (93.1)	95 (92.2)	
**After leakage**	59 (6.9)	8 (7.8)	
**Stoma type**			0.494
**Ileostomy**	135 (15.8)	19 (18.4)	
**Colostomy**	718 (84.2)	84 (81.6)	
**CVA (%)**	23 (2.7)	6 (5.8)	0.08
**Liver cirrhosis (%)**	11 (1.3)	3 (2.9)	0.195
T stage (%)			0.005
0–2	265 (31.1)	19 (18.4)	
3	533 (62.5)	71 (68.9)	
4	55 (6.4)	13 (12.6)	
**N stage (%)**			0.001
**0**	482 (56.5)	42 (40.8)	
**1**	233 (27.3)	30 (29.1)	
**2**	138 (16.2)	31 (30.1)	
**Low rectal tumor (%)**	354 (41.5)	46 (44.7)	0.539
**Involved circumferential margin (%)**	258 (30.3)	51 (49.5)	< 0.001
**Laparoscopy (%)**	281 (32.9)	27 (26.2)	0.167
**Albumin < 3.5 g/dL (%)**	48 (5.6)	10 (9.7)	0.101
**CEA > 5** ng/mL	183 (21.5)	40 (38.8)	< 0.001
**Neoadjuvant RT**^a^** (%)**	261 (30.6)	25 (24.3)	0.185
**Adjuvant chemotherapy**	260 (30.5)	19 (18.4)	0.011
**Chemotherapy with Oxaliplatin**^b^	54 (20.8)	8 (42.1)	0.031

*BMI* body mass index, *eGFR* estimated Glomerular filtration rate, *CVA* Cerebrovascular accident, *CEA* carcinoembryonic antigen, *RT* radiotherapy

^a^Including both long-course and short-course radiation therapy

^b^Percentage of chemotherapy patients

In the multivariate survival analysis for stoma non-closure (Table 4), age above 80, anastomotic leakage and the N2 stage emerged as risk factors for stoma non-closure (OR:3.53, 4.09, 5.35 respectively, *p* < 0.001). Instead, adjuvant chemotherapy was identified as a protective factor with an odds ratio of 0.258 (*p* < 0.001) after backward selection.

**Table 4:** Univariate and multivariate analysis for temporary stoma non-closure

	**Before backward selection**		**After backward selection**		
	OR	*p*-value	OR	*p*-value	95% CI
**Age > 80**	3.53	< 0.001	3.56	< 0.001	1.86–6.78
**Male Sex**	1.55	0.093	1.59	0.072	0.96–2.64
**Anastomotic leakage**	4.09	< 0.001	4.10	< 0.001	2.34–7.53
**T stage**					
**0–2**	1				
**3**	1.43	0.268			
**4**	1.87	0.190			
**N stage**					
**0**	1		1		
**1**	2.26	0.005	2.41	0.002	1.39–4.19
**2**	4.78	< 0.001	5.35	< 0.001	2.90–9.87
**Low rectal tumor**	1.96	0.006	1.83	0.011	1.15–2.92
**Adjuvant chemotherapy**	0.25	< 0.001	0.258	< 0.001	0.14–4.78
**Neoadjuvant Radiotherapy**	0.81	0.416			
**CEA ≥ 5**	1.71	0.032	1.85	0.011	1.15–2.98
**Involved CM**	1.94	0.005	2.12	0.001	1.35–3.32
**Alb < 3.5**	1.92	0.111	2.08	0.069	0.95–4.58
**eGFR < 30**	2.90	0.041	3.05	0.029	1.12–8.29
**CVA**	1.45	0.500			
**Cirrhosis**	1.77	0.468			

*CEA* carcinoembryonic antigen, *CM* circumferential margin, *eGFR* estimated Glomerular filtration rate, *CVA Cerebrovascular accident*

**Fig. 2:**
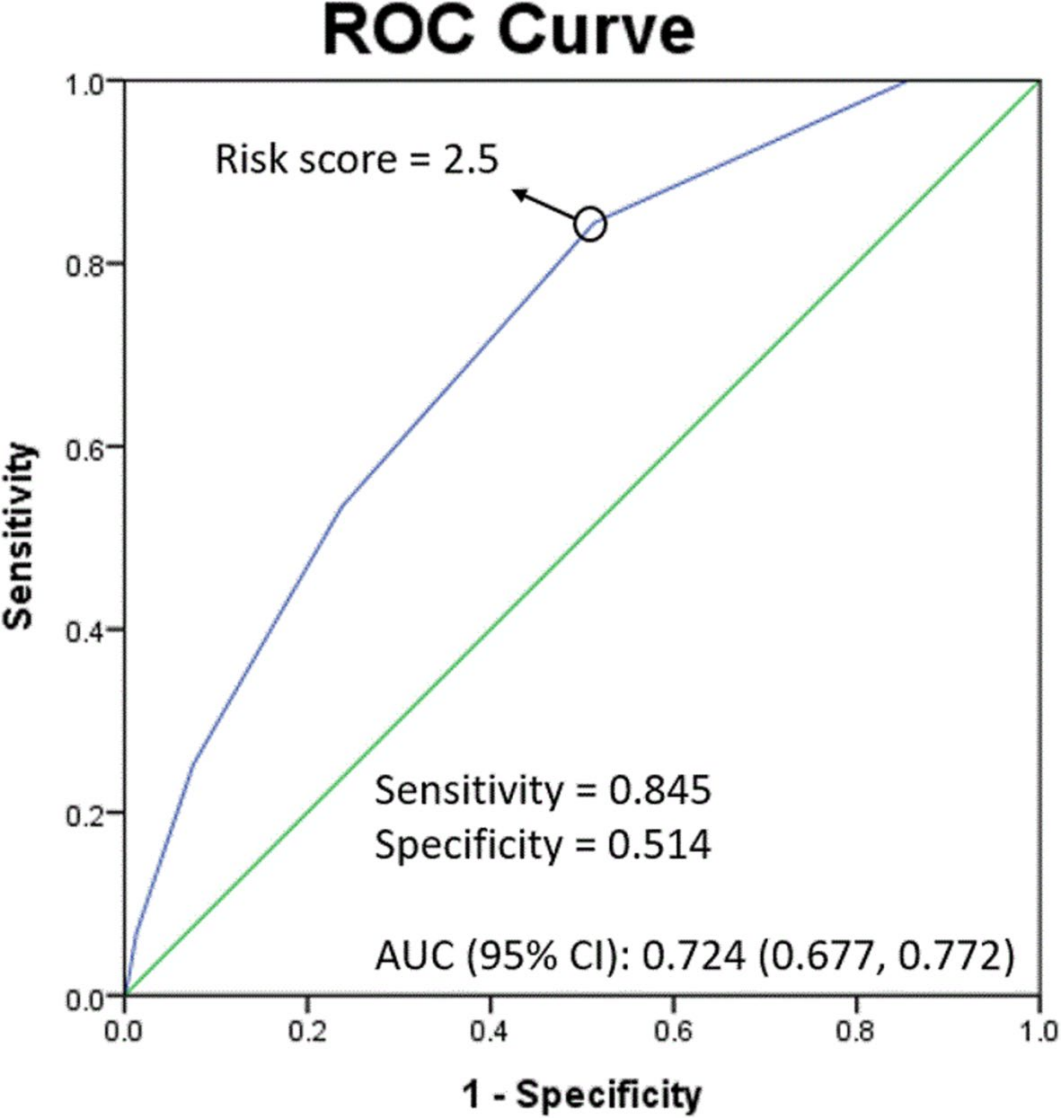
ROC Curve for Temporary Stoma Non-Closure Risk. The Receiver Operating Characteristic (ROC) curve analysis for the risk of non-closure of temporary stomas indicates a cutoff value of 2.5. This is associated with a sensitivity of 84.5% and a specificity of 51.4%. The area under the curve (AUC) stands at 0.724

**Fig. 3:**
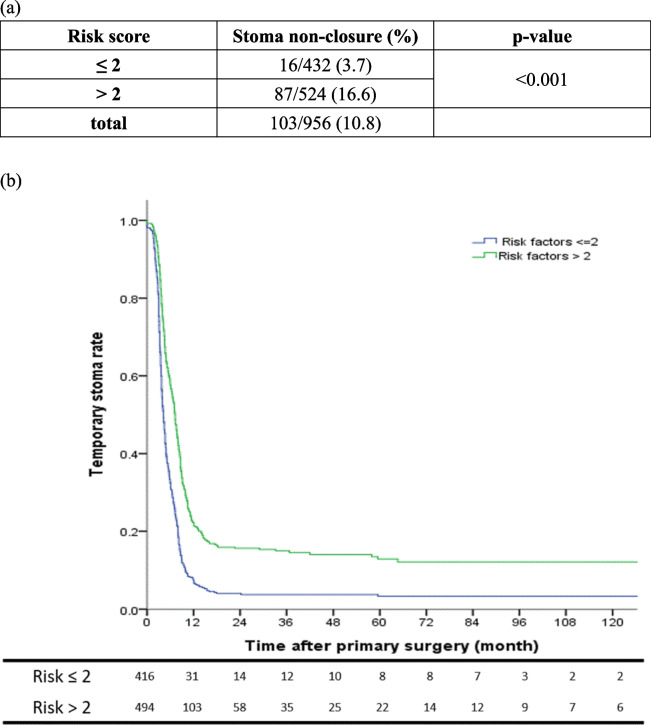
(**a**) Preoperative risk stratification of a temporary stoma non-closure, (**b**) stoma non-closure rate between risk score ≤ 2 and > 2

Based on these insights, advanced age, anastomotic leakage, N stage, lower rectal cancer presence, serum CEA > 5 ng/mL, tumors involving circumferential margins, and eGFR ≤ 30 mL/min/1.73m^2^ were highlighted for risk stratification. The ROC curve analysis for variables associated with stoma non-closure indicated a risk cutoff value of 2.5, with a sensitivity of 84.5% and specificity of 51.4% (Fig. 2). The area under the curve (AUC) stood at 0.724 (95% CI: 0.677- 0.772). Furthermore, the stoma non-closure rate was 16.6% in patients with a risk score above 2, significantly higher than those with a risk score of 2 or below (3.7% vs. 16.6%, *p* < 0.001). (Fig. 3).

## Discussion

This study aimed to investigate the risk factors associated with the non-closure of initially intended temporary stomas, which could not undergo stoma reversal surgery. While previous studies primarily discussed preoperative or post-operative risks of permanent stomas, our focus was on identifying factors that contribute to the non-closure of stomas. In this study, the rate of stoma non-closure was 10.7%. Existing research has indicated that the rate of permanent stomas, including re-do stomas and stoma non-closures, ranged from 3% to 23.2% [9–12], and the rate of non-closure for temporary stomas ranged from 3 to 25% [10, 12, 16, 9, 11]. We identified seven independent risk factors for stoma non-closure, namely advanced age, anastomotic leakage, N stages, tumor distance from the anal verge < 5 cm, preoperative CEA level > 5, and involved circumferential margin, and eGFR ≤ 30 mL/min/1.73 m^2^. These factors can be assessed and communicated to patients by surgeons before the operation.

Cancer progression involving local recurrence and distant metastasis has been identified as the primary cause of permanent stomas [13, 9, 11, 14]. In our study, cancer recurrence accounted for most stoma non-closures (35%), followed by complications related to anastomosis (22.3%). Den Dulk et al. identified advanced age, creation of a secondary stoma, diverting ostomy, surgical difficulties, and cancer recurrence as factors limiting stoma reversal [10]. Lindgren et al. reported that 56% of patients with symptomatic anastomotic leakage ended up with a permanent stoma [12]. Our analysis revealed that stenosis and leakage accounted for more than half of the cases with anastomosis-related complications. The inflammatory response triggered by postoperative infection, leading to increased production of IL-6 and VEGF, is considered one of the mechanisms contributing to anastomotic leakage [21]. Thus, the management of postoperative anastomotic complications remains a significant challenge for surgeons to address.

Due to the lower BMI of our patient population, loop colostomy was primarily chosen as the type of diverting procedure. Although ileostomy is considered a less invasive and more comfortable procedure compared to colostomy, the digestive enzymes present in the output can irritate the mucosa and skin after undergoing ileostomy. The high volume of stoma output may cause dehydration and electrolyte imbalance. In contrast, colostomies produce stool without digestive enzymes. Colostomy patients can be reassured that there are no strict dietary restrictions. Recent studies have demonstrated the comparable diverting function and post-ostomy mortality rates between colostomies and ileostomies [22, 23]. In our study, the proportion of stoma non-closure did not show statistical significance between colostomies and ileostomies (81.6% vs. 18.4%, *p* = 0.494). Therefore, the selection of colostomy or ileostomy should still be individually considered based on the patient's specific condition.

Preoperative serum CEA levels have been found to influence the reversal of stomas. Our previous study reported that the preoperative CEA level was an independent prognostic factor for stages I-III CRC after curative resection, particularly when the CEA level > 10 ng/ml [24]. Another study demonstrated that elevated preoperative serum CEA levels in patients with stage 0-III mid-to-low rectal cancer who underwent curative low anterior resection were related to a higher rate of permanent stomas and poor oncological prognosis [19]. Our current study found that a CEA level above 5 ng/ml was associated with a higher rate of stoma non-closure (38.8%). Even after neoadjuvant chemoradiation therapy, persistently elevated CEA levels can lead to cancer progression with increased perianal invasion, resulting in reduced overall and disease-free survival. In conclusion, monitoring the pre-treatment CEA level is essential and serves as a predictive factor for stoma non-closure.

Circumferential margins have been identified as an independent prognostic factor in rectal cancer surgery. Liu et al. demonstrated that a circumferential margin less than 1 mm was associated with an increased cancer-specific mortality rate [25]. Involvement of the circumferential margin raises the risk of local recurrence and increases the need for permanent stomas [17]. In our study, patients with non-closure of stomas had a higher rate of involved circumferential margins (49.5%). Consequently, surgeons must strive to achieve maximum circumferential resection margins to improve outcomes and reverse stoma.

Impaired renal function is associated to temporary stoma non-closure. A retrospective study demonstrated that a decreased eGFR of ≤ 45 mL/min/1.73 m^2^ was an independent predictor of non-closure in patients who underwent acute resection of left-sided obstructive colon cancer [18]. Our study revealed that eGFR ≤ 30 mL/min/1.73 m^2^ was a negative predicting factor of reversal of stoma. Patients with an impaired preoperative renal function are less motivated for stoma reversal due to a higher operation risk [26]. Due to the complication rate of LAR itself, which can be as high as 20% [27], for patients with a higher perioperative risk, such as poor renal function, it is crucial to evaluate and inform them of the risk of being unable to undergo subsequent stoma reversal surgery.

Neoadjuvant concurrent therapy and radiotherapy in advanced rectal cancer patients remains a controversial issue. Zhou et al. demonstrated that radiotherapy and chemotherapy were not correlated with the non-closure of dysfunctional stomas and could reduce the local recurrence rate in lower rectal cancer [14, 28, 29]. Nevertheless, while neoadjuvant chemoradiation therapy and adjuvant radiotherapy can downstage colorectal cancer by reducing tumor size, preoperative radiotherapy may increase the risk of complications such as anastomotic leakage, leading to the need for a permanent stoma [30, 31]. A multivariate analysis reported that preoperative radiotherapy can cause an irreversible secondary stoma [10]. Our study, however, showed no significant relationship between neoadjuvant radiotherapy and stoma non-closure. A longitudinal study conducted by Junginger et al. revealed that the application of long-course neoadjuvant chemoradiation therapy resulted in a reduction of tumor-related permanent stomas. However, it led to an increase in anastomosis-related permanent stomas. Consequently, the overall permanent stoma rate remained unchanged at 16% compared to 19% [13]. This study and our report show that current chemoradiation therapy has limited advantage in improving organ preservation. Furthermore, proton beam therapy is less invasive for rectal cancer compared to photon-based radiotherapy [32]. Therefore, the application of proton therapy in rectal cancer may have the potential benefit of improving tumor control and organ preservation Additionally, adjuvant chemotherapy was identified as a protective factor against stoma non-closure (OR: 0.26, *p* < 0.001). In many studies, including our reports, cancer recurrence is identified as one of the main factors leading to permanent stoma use [9, 11, 13, 14]. For rectal cancer patients with yp stage II/III, adjuvant FOLFOX after neoadjuvant chemoradiation and curative resection can improve disease-free survival [33]. Therefore, if the patients with locally advanced staging rectal cancer and is unavailable of receiving adjuvant chemotherapy, temporary stoma may have a higher risk of non-closure due to cancer recurrence.

This research has several limitations that should be acknowledged. Firstly, the study's retrospective design and single-center introduce the possibility of selection bias. The sample size was limited and consisted of patients from a single ethnic group, which may affect the generalizability of the findings to other populations. Secondly, the neoadjuvant radiotherapy course and surgical procedures varied due to surgeon discretion and patient preferences, leading to unavoidable differences. Additionally, the use of diverse regimens, including radiotherapy or combined chemoradiotherapy, contributed to the heterogeneous treatment approaches. Thirdly, the accuracy of restaging by MRI examination may be compromised, particularly after neoadjuvant therapy, potentially leading to misdiagnosis [19]. To mitigate this, we relied on the pathological staging method as an alternative to evaluate the extent of the cancer more precisely. Lastly, deviations from standardized treatment protocols may introduce variability and confounding factors. These limitations should be considered when interpreting findings for clinical practice. Future research with larger, multicenter cohorts and more comprehensive evaluations is warranted to overcome these limitations and provide more robust evidence.

## Conclusions

Advanced age, symptomatic anastomotic leakage, positive nodal status, preoperative serum CEA levels > 5 ng/ml, circumferential margins involvement, and eGFR ≤ 30 mL/min/1.73m^2^ are independent prognostic factors for temporary stoma non-closure in rectal cancer patients. The non-closure rate may increase to 16.6% when more than 2 factors are presented. Surgeons should thoroughly evaluate and inform the patients before surgery.

## Data Availability

No datasets were generated or analysed during the current study.

## Abbreviations

CEA: Carcinoembryonic antigen
CCRT: Concurrent chemoradiation
ROC: Receiver operating characteristic
RT: Radiotherapy
AUC: Area under the curve
